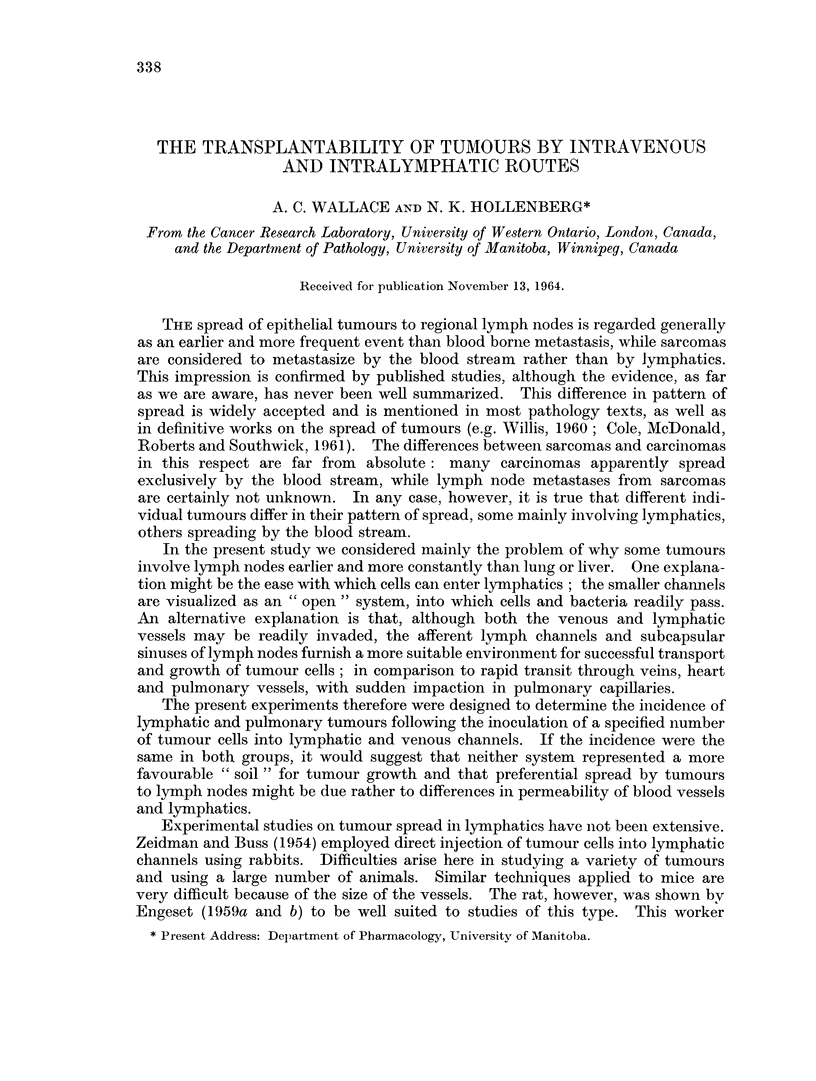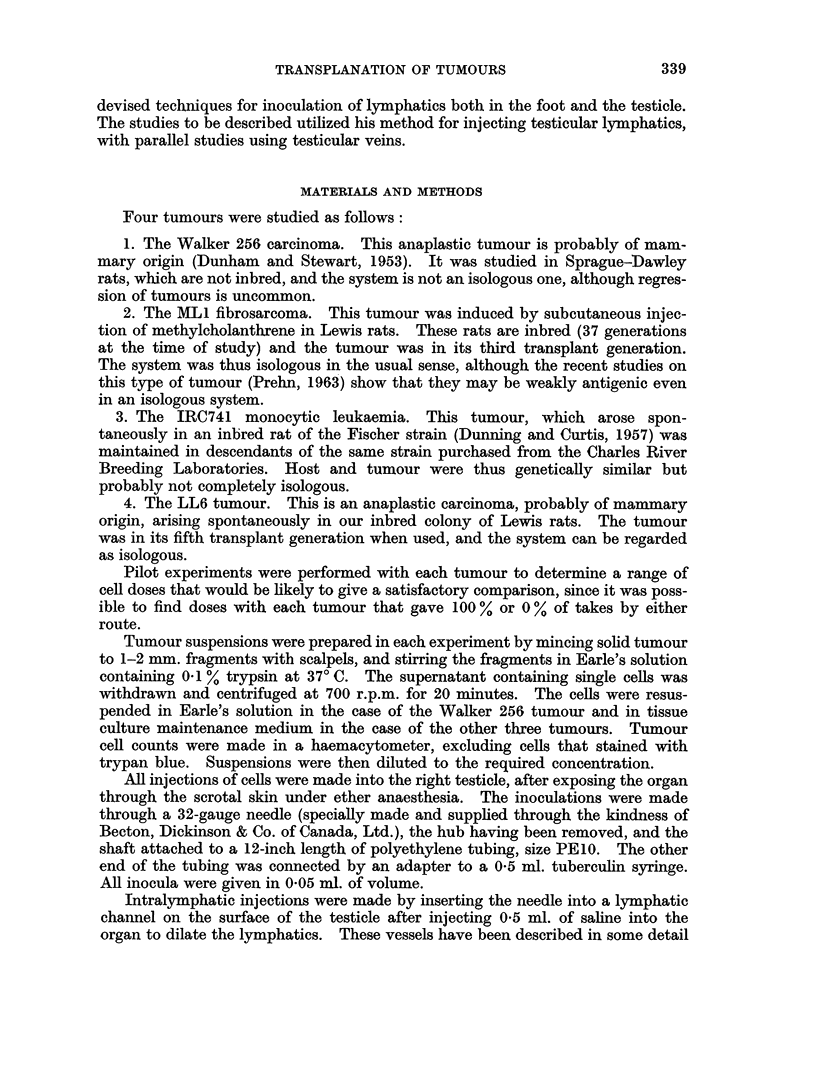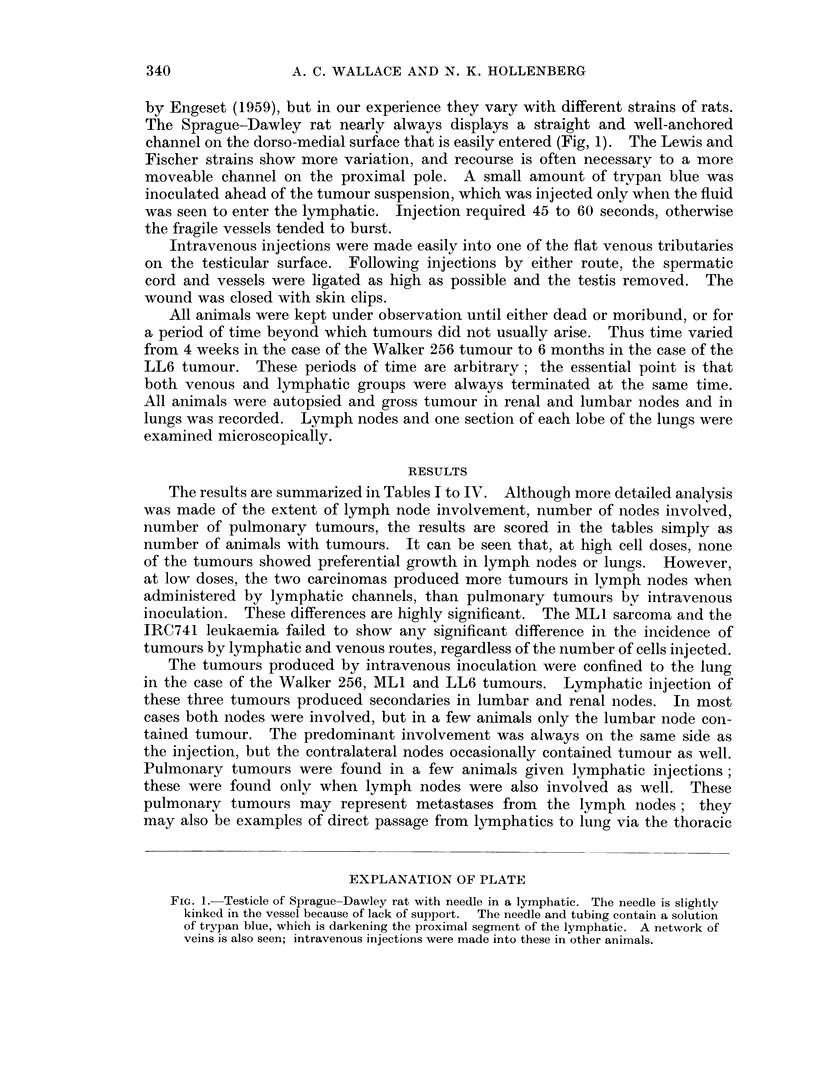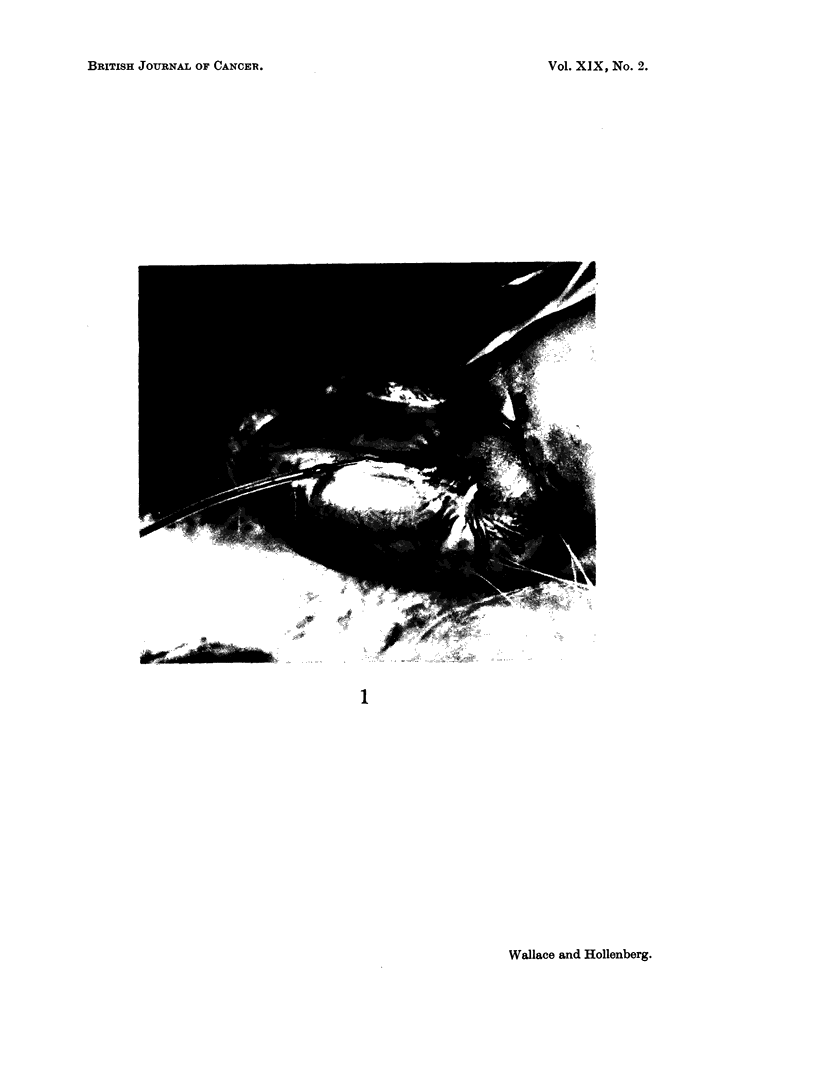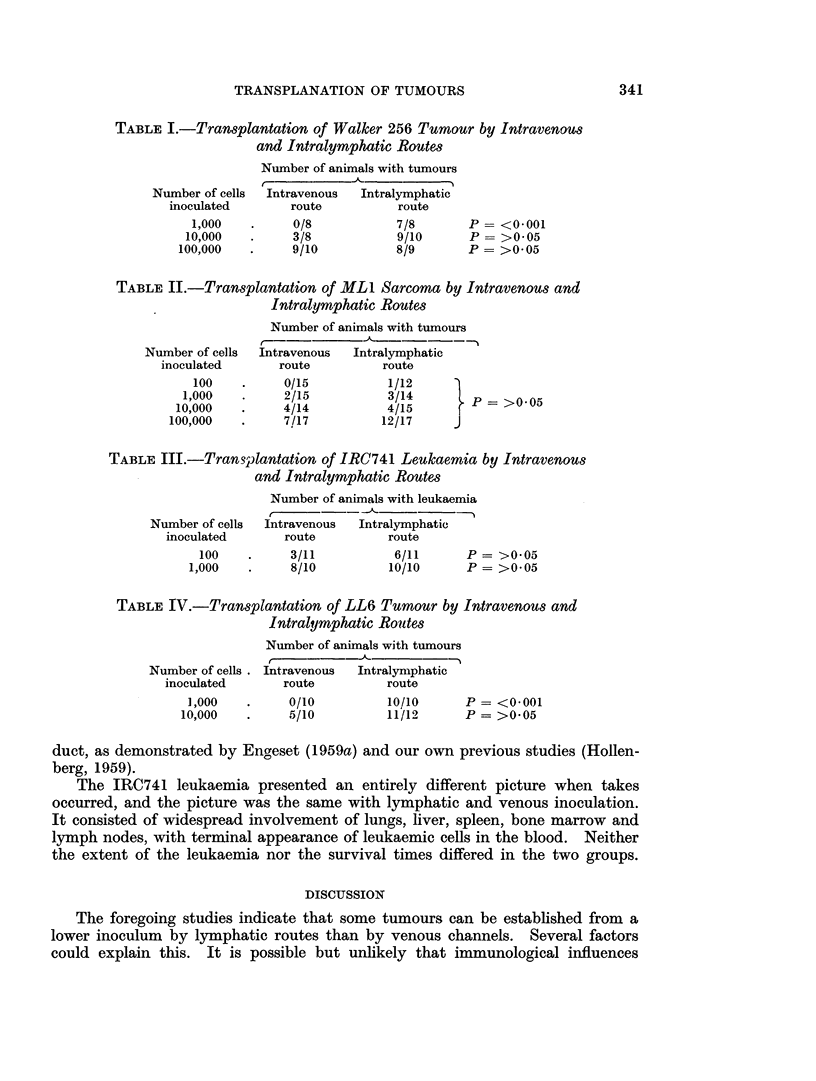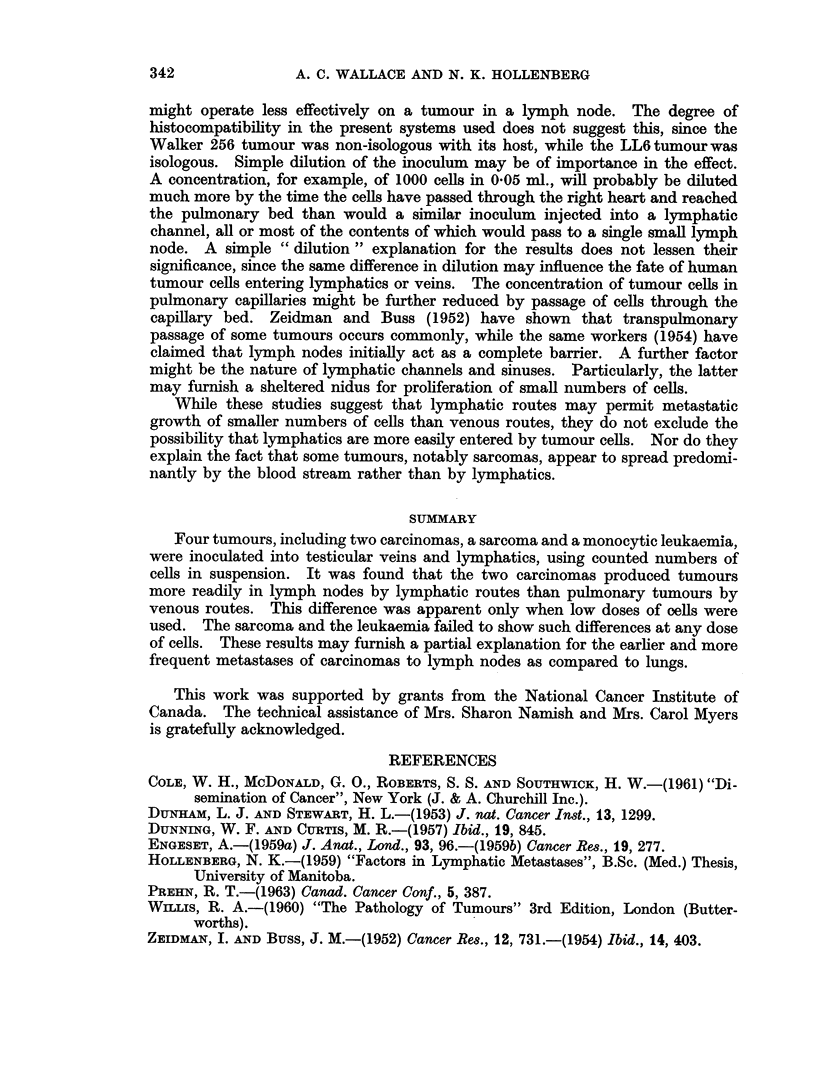# The Transplantability of Tumours by Intravenous and Intralymphatic Routes

**DOI:** 10.1038/bjc.1965.40

**Published:** 1965-06

**Authors:** A. C. Wallace, N. K. Hollenberg

## Abstract

**Images:**


### THE TRANSPLANTABILITY OF TUMOURS BY INTRAVENOUS AND INTRALYMPHATIC ROUTES

A. C. WALLACE AND N. K. HOLLENBERG*

*From the Cancer Research Laboratory, University of Western Ontario, London, Canada, and the Department of Pathology, University of Manitoba, Winnipeg, Canada*

Received for publication November 13, 1964.

THE spread of epithelial tumours to regional lymph nodes is regarded generally as an earlier and more frequent event than blood borne metastasis, while sarcomas are considered to metastasize by the blood stream rather than by lymphatics. This impression is confirmed by published studies, although the evidence, as far as we are aware, has never been well summarized. This difference in pattern of spread is widely accepted and is mentioned in most pathology texts, as well as in definitive works on the spread of tumours (e.g. Willis, 1960 ; Cole, McDonald, Roberts and Southwick, 1961). The differences between sarcomas and carcinomas in this respect are far from absolute : many carcinomas apparently spread exclusively by the blood stream, while lymph node metastases from sarcomas are certainly not unknown. In any case, however, it is true that different individual tumours differ in their pattern of spread, some mainly involving lymphatics, others spreading by the blood stream.

In the present study we considered mainly the problem of why some tumours involve lymph nodes earlier and more constantly than lung or liver. One explanation might be the ease with which cells can enter lymphatics ; the smaller channels are visualized as an " open " system, into which cells and bacteria readily pass. An alternative explanation is that, although both the venous and lymphatic vessels may be readily invaded, the afferent lymph channels and subcapsular sinuses of lymph nodes furnish a more suitable environment for successful transport and growth of tumour cells ; in comparison to rapid transit through veins, heart and pulmonary vessels, with sudden impaction in pulmonary capillaries.

The present experiments therefore were designed to determine the incidence of lymphatic and pulmonary tumours following the inoculation of a specified number of tumour cells into lymphatic and venous channels. If the incidence were the same in both groups, it would suggest that neither system represented a more favourable " soil " for tumour growth and that preferential spread by tumours to lymph nodes might be due rather to differences in permeability of blood vessels and lymphatics.

Experimental studies on tumour spread in lymphatics have not been extensive. Zeidman and Buss (1954) employed direct injection of tumour cells into lymphatic channels using rabbits. Difficulties arise here in studying a variety of tumours and using a large number of animals. Similar techniques applied to mice are very difficult because of the size of the vessels. The rat, however, was shown by Engeset (1959*a* and *b*) to be well suited to studies of this type. This worker

* Present Address: Department of Pharmacology, University of Manitoba.

devised techniques for inoculation of lymphatics both in the foot and the testicle. The studies to be described utilized his method for injecting testicular lymphatics, with parallel studies using testicular veins.

### MATERIALS AND METHODS

Four tumours were studied as follows :

1. The Walker 256 carcinoma. This anaplastic tumour is probably of mammary origin (Dunham and Stewart, 1953). It was studied in Sprague–Dawley rats, which are not inbred, and the system is not an isologous one, although regression of tumours is uncommon.
2. The ML1 fibrosarcoma. This tumour was induced by subcutaneous injection of methylcholanthrene in Lewis rats. These rats are inbred (37 generations at the time of study) and the tumour was in its third transplant generation. The system was thus isologous in the usual sense, although the recent studies on this type of tumour (Prehn, 1963) show that they may be weakly antigenic even in an isologous system.
3. The IRC741 monocytic leukaemia. This tumour, which arose spontaneously in an inbred rat of the Fischer strain (Dunning and Curtis, 1957) was maintained in descendants of the same strain purchased from the Charles River Breeding Laboratories. Host and tumour were thus genetically similar but probably not completely isologous.
4. The LL6 tumour. This is an anaplastic carcinoma, probably of mammary origin, arising spontaneously in our inbred colony of Lewis rats. The tumour was in its fifth transplant generation when used, and the system can be regarded as isologous.

Pilot experiments were performed with each tumour to determine a range of cell doses that would be likely to give a satisfactory comparison, since it was possible to find doses with each tumour that gave 100% or 0% of takes by either route.

Tumour suspensions were prepared in each experiment by mincing solid tumour to 1–2 mm. fragments with scalpels, and stirring the fragments in Earle's solution containing 0·1% trypsin at 37° C. The supernatant containing single cells was withdrawn and centrifuged at 700 r.p.m. for 20 minutes. The cells were resuspended in Earle's solution in the case of the Walker 256 tumour and in tissue culture maintenance medium in the case of the other three tumours. Tumour cell counts were made in a haemacytometer, excluding cells that stained with trypan blue. Suspensions were then diluted to the required concentration.

All injections of cells were made into the right testicle, after exposing the organ through the scrotal skin under ether anaesthesia. The inoculations were made through a 32-gauge needle (specially made and supplied through the kindness of Becton, Dickinson & Co. of Canada, Ltd.), the hub having been removed, and the shaft attached to a 12-inch length of polyethylene tubing, size PE10. The other end of the tubing was connected by an adapter to a 0·5 ml. tuberculin syringe. All inocula were given in 0·05 ml. of volume.

Intralymphatic injections were made by inserting the needle into a lymphatic channel on the surface of the testicle after injecting 0·5 ml. of saline into the organ to dilate the lymphatics. These vessels have been described in some detail by Engeset (1959), but in our experience they vary with different strains of rats. The Sprague–Dawley rat nearly always displays a straight and well-anchored channel on the dorso-medial surface that is easily entered (Fig, 1). The Lewis and Fischer strains show more variation, and recourse is often necessary to a more moveable channel on the proximal pole. A small amount of trypan blue was inoculated ahead of the tumour suspension, which was injected only when the fluid was seen to enter the lymphatic. Injection required 45 to 60 seconds, otherwise the fragile vessels tended to burst.

Intravenous injections were made easily into one of the flat venous tributaries on the testicular surface. Following injections by either route, the spermatic cord and vessels were ligated as high as possible and the testis removed. The wound was closed with skin clips.

All animals were kept under observation until either dead or moribund, or for a period of time beyond which tumours did not usually arise. Thus time varied from 4 weeks in the case of the Walker 256 tumour to 6 months in the case of the LL6 tumour. These periods of time are arbitrary ; the essential point is that both venous and lymphatic groups were always terminated at the same time. All animals were autopsied and gross tumour in renal and lumbar nodes and in lungs was recorded. Lymph nodes and one section of each lobe of the lungs were examined microscopically.

### RESULTS

The results are summarized in Tables I to IV. Although more detailed analysis was made of the extent of lymph node involvement, number of nodes involved, number of pulmonary tumours, the results are scored in the tables simply as number of animals with tumours. It can be seen that, at high cell doses, none of the tumours showed preferential growth in lymph nodes or lungs. However, at low doses, the two carcinomas produced more tumours in lymph nodes when administered by lymphatic channels, than pulmonary tumours by intravenous inoculation. These differences are highly significant. The ML1 sarcoma and the IRC741 leukaemia failed to show any significant difference in the incidence of tumours by lymphatic and venous routes, regardless of the number of cells injected.

The tumours produced by intravenous inoculation were confined to the lung in the case of the Walker 256, ML1 and LL6 tumours. Lymphatic injection of these three tumours produced secondaries in lumbar and renal nodes. In most cases both nodes were involved, but in a few animals only the lumbar node contained tumour. The predominant involvement was always on the same side as the injection, but the contralateral nodes occasionally contained tumour as well. Pulmonary tumours were found in a few animals given lymphatic injections ; these were found only when lymph nodes were also involved as well. These pulmonary tumours may represent metastases from the lymph nodes ; they may also be examples of direct passage from lymphatics to lung via the thoracic

---

EXPLANATION OF PLATE

FIG. 1.—Testicle of Sprague–Dawley rat with needle in a lymphatic. The needle is slightly kinked in the vessel because of lack of support. The needle and tubing contain a solution of trypan blue, which is darkening the proximal segment of the lymphatic. A network of veins is also seen; intravenous injections were made into these in other animals.

1

Wallace and Hollenberg.

TABLE I.—*Transplantation of Walker 256 Tumour by Intravenous and Intralymphatic Routes*

| Number of cells inoculated | Number of animals with tumours: Intravenous route | Intralymphatic route | |
|---|---|---|---|
| 1,000 | 0/8 | 7/8 | P = <0·001 |
| 10,000 | 3/8 | 9/10 | P = >0·05 |
| 100,000 | 9/10 | 8/9 | P = >0·05 |

TABLE II.—*Transplantation of ML1 Sarcoma by Intravenous and Intralymphatic Routes*

| Number of cells inoculated | Number of animals with tumours: Intravenous route | Intralymphatic route | |
|---|---|---|---|
| 100 | 0/15 | 1/12 | P = >0·05 |
| 1,000 | 2/15 | 3/14 | |
| 10,000 | 4/14 | 4/15 | |
| 100,000 | 7/17 | 12/17 | |

TABLE III.—*Transplantation of IRC741 Leukaemia by Intravenous and Intralymphatic Routes*

| Number of cells inoculated | Number of animals with leukaemia: Intravenous route | Intralymphatic route | |
|---|---|---|---|
| 100 | 3/11 | 6/11 | P = >0·05 |
| 1,000 | 8/10 | 10/10 | P = >0·05 |

TABLE IV.—*Transplantation of LL6 Tumour by Intravenous and Intralymphatic Routes*

| Number of cells inoculated | Number of animals with tumours: Intravenous route | Intralymphatic route | |
|---|---|---|---|
| 1,000 | 0/10 | 10/10 | P = <0·001 |
| 10,000 | 5/10 | 11/12 | P = >0·05 |

duct, as demonstrated by Engeset (1959*a*) and our own previous studies (Hollenberg, 1959).

The IRC741 leukaemia presented an entirely different picture when takes occurred, and the picture was the same with lymphatic and venous inoculation. It consisted of widespread involvement of lungs, liver, spleen, bone marrow and lymph nodes, with terminal appearance of leukaemic cells in the blood. Neither the extent of the leukaemia nor the survival times differed in the two groups.

### DISCUSSION

The foregoing studies indicate that some tumours can be established from a lower inoculum by lymphatic routes than by venous channels. Several factors could explain this. It is possible but unlikely that immunological influences might operate less effectively on a tumour in a lymph node. The degree of histocompatibility in the present systems used does not suggest this, since the Walker 256 tumour was non-isologous with its host, while the LL6 tumour was isologous. Simple dilution of the inoculum may be of importance in the effect. A concentration, for example, of 1000 cells in 0·05 ml., will probably be diluted much more by the time the cells have passed through the right heart and reached the pulmonary bed than would a similar inoculum injected into a lymphatic channel, all or most of the contents of which would pass to a single small lymph node. A simple "dilution" explanation for the results does not lessen their significance, since the same difference in dilution may influence the fate of human tumour cells entering lymphatics or veins. The concentration of tumour cells in pulmonary capillaries might be further reduced by passage of cells through the capillary bed. Zeidman and Buss (1952) have shown that transpulmonary passage of some tumours occurs commonly, while the same workers (1954) have claimed that lymph nodes initially act as a complete barrier. A further factor might be the nature of lymphatic channels and sinuses. Particularly, the latter may furnish a sheltered nidus for proliferation of small numbers of cells.

While these studies suggest that lymphatic routes may permit metastatic growth of smaller numbers of cells than venous routes, they do not exclude the possibility that lymphatics are more easily entered by tumour cells. Nor do they explain the fact that some tumours, notably sarcomas, appear to spread predominantly by the blood stream rather than by lymphatics.

### SUMMARY

Four tumours, including two carcinomas, a sarcoma and a monocytic leukaemia, were inoculated into testicular veins and lymphatics, using counted numbers of cells in suspension. It was found that the two carcinomas produced tumours more readily in lymph nodes by lymphatic routes than pulmonary tumours by venous routes. This difference was apparent only when low doses of cells were used. The sarcoma and the leukaemia failed to show such differences at any dose of cells. These results may furnish a partial explanation for the earlier and more frequent metastases of carcinomas to lymph nodes as compared to lungs.

This work was supported by grants from the National Cancer Institute of Canada. The technical assistance of Mrs. Sharon Namish and Mrs. Carol Myers is gratefully acknowledged.

### REFERENCES

Cole, W. H., McDonald, G. O., Roberts, S. S. and Southwick, H. W.—(1961) "Dissemination of Cancer", New York (J. & A. Churchill Inc.).
Dunham, L. J. and Stewart, H. L.—(1953) *J. nat. Cancer Inst.*, **13**, 1299.
Dunning, W. F. and Curtis, M. R.—(1957) *Ibid.*, **19**, 845.
Engeset, A.—(1959*a*) *J. Anat., Lond.*, **93**, 96.—(1959*b*) *Cancer Res.*, **19**, 277.
Hollenberg, N. K.—(1959) "Factors in Lymphatic Metastases", B.Sc. (Med.) Thesis, University of Manitoba.
Prehn, R. T.—(1963) *Canad. Cancer Conf.*, **5**, 387.
Willis, R. A.—(1960) "The Pathology of Tumours" 3rd Edition, London (Butterworths).
Zeidman, I. and Buss, J. M.—(1952) *Cancer Res.*, **12**, 731.—(1954) *Ibid.*, **14**, 403.